# Association Between Exercise Frequency and Self-Reported Endoscopy-Related Musculoskeletal Symptoms Among Endoscopists

**DOI:** 10.7759/cureus.109315

**Published:** 2026-05-20

**Authors:** Sankirth Madabhushi, Garrick Gu, Sanjay Rau, Peter Sullivan, Matthew Petersile

**Affiliations:** 1 Medicine, University of Massachusetts, Worcester, USA; 2 Gastroenterology, University of Massachusetts, Worcester, USA

**Keywords:** endoscopy-related injury, ergonomics, exercise, occupational health risk, work related injuries

## Abstract

Background

Endoscopy-related injuries (ERIs) are common among endoscopists and can lead to decreased procedural volume, disability, and early retirement. The impact of physical conditioning on endoscopy-related musculoskeletal symptoms is not well studied. We aimed to investigate the association between exercise and the prevalence of self-reported endoscopy-related musculoskeletal symptoms (SERMS).

Methods

We conducted a cross-sectional survey of gastroenterology fellows and attendings at a tertiary-care academic center. Participants reported demographics, procedural volume, ergonomics training history, injury history, and exercise frequency and type. Exercise frequency was further categorized into fewer than four days/week or greater than or equal to four days/week of moderate-to-strenuous activity. Statistical analysis utilized Fisher’s exact and Mann-Whitney U tests.

Results

Of the 25 respondents (median age 37.5 years), 18 (72%) reported a history of SERMS. Endoscopists with fewer than or equal to six years of experience reported significantly higher rates of thumb pain (9 [69%] vs. 3 [25%], p = 0.047) and hand pain (6 [46%] vs. 0 [0%], p = 0.015) compared to established physicians. Physicians exercising at least four days per week had a significantly lower prevalence of thumb pain compared to those exercising less than four days (3 [23%] vs. 9 [75%]; p = 0.017). There were no significant associations between injury rates and procedural volume or prior ergonomics training.

Conclusion

ERIs are highly prevalent, yet data regarding ergonomics and injury prevention are limited. In this study, frequent moderate-to-strenuous exercise was associated with decreased prevalence of self-reported thumb pain related to performing endoscopy. These findings support the need for further research on protective factors for SERMS and perhaps the institution of structured physical conditioning programs for endoscopist occupational health.

## Introduction

The American Society of Gastrointestinal Endoscopy (ASGE) guidelines state that endoscopy-related injuries (ERIs) are musculoskeletal injuries (bothersome pain or numbness) caused by repetitive microtrauma to the connective tissues of the body and that the most common sites of ERI are hands and fingers, back, and neck [1]. ERIs may occur, as performing endoscopy involves navigating complex anatomy and standing for extended durations during endoscopic procedures [2]. ERIs among endoscopists have been self-reported to be as high as 90%, with the most common injuries affecting the hands, wrists, neck, and back [2]. Short-term logistical complications include slower procedure times, decreased procedural volume, and delays in patient care. There are long-term implications as well, including disability and earlier retirement, further compounding the existing shortage of gastroenterologists [3]. Prior studies identified risks for ERIs, including a higher procedural volume, increased procedural time, small hand size, female gender, and age [1].

Endoscopic procedural volume and complexity have increased dramatically over the last two decades, resulting in high rates of ERIs among endoscopists. Despite the rise in ERIs, ergonomic curricula are uncommon at most training institutions [1]. Furthermore, although the prevalence of ERIs has been assessed in a few recent studies, research into safe practices and protective factors is lacking in this domain [1]. Performing endoscopy involves high, repetitive loads on the upper extremity and requires sustained postural stabilization, particularly involving the thumb and hands. Prior ergonomics and occupational health literature endorse movement-based interventions, such as microbreaks and stretching. Thus, exercise may have a plausible role in improving neuromuscular endurance relevant to endoscopic tasks [4].

This study aims to explore associations between exercise and self-reported endoscopy-related musculoskeletal symptoms (SERMS) rates and provide a foundation for future research on this subject. Primarily, we investigated whether frequent, moderate-to-strenuous exercise was associated with particular SERMS.

This article was previously presented as a poster during Digestive Disease Week on May 3, 2025.

## Materials and methods

Study design

We planned an observational cross-sectional study at a large tertiary-care academic medical center designed to include gastroenterology fellows and attendings at our institution who performed endoscopy. This approach was selected so that the endoscopists included were exposed to the same uniform institutional protocols to minimize environmental confounding. Only respondents who performed endoscopy were permitted to proceed, while those without endoscopy experience had their survey terminated after answering a mandatory screening question. An electronic self-administered survey was designed and distributed via email between August and September 2024 using REDCap (Research Electronic Data Capture, Vanderbilt University, Nashville, TN) and asked questions addressing demographics, exercise frequency, endoscopic practices, and injury history [5,6]. All responses were anonymous. Survey items were modeled after the survey used by Pawa et al. in their study of ERIs incorporated in the ASGE guidelines [1,2].

Survey content

Our survey asked participants about demographics (gender, height, weight, age), with the option to prefer not to answer. Regarding endoscopic practices, participants were asked to self-approximate the volume of procedures performed per week, years of experience, and completion of any ergonomics training. To align with our survey-based design, we evaluated self-reported endoscopy-related musculoskeletal symptoms, defined as bothersome pain or numbness perceived by participants as related to their endoscopic practice, rather than clinical or structural diagnoses (ERIs) [1,2]. Participants were asked, "Have you ever experienced or are you currently experiencing injury (bothersome pain or numbness) in your neck, back, upper, or lower limbs that you attributed to performing endoscopy?" To explore SERMS, participants were asked to select all that apply from a comprehensive checklist covering 14 distinct sites (thumb pain, hand pain, hand numbness, carpal tunnel syndrome, De Quervain's tenosynovitis, wrist pain, elbow pain, shoulder pain, neck pain, upper back pain, lower back pain, hip pain, knee pain, and foot pain) and were asked to specify the side affected (right, left, or both). Finally, we examined the role of exercise by asking participants to describe the types of exercise they performed and how many days per week they engaged in moderate-to-strenuous exercise. Physical activity was classified based on the American Heart Association's (AHA) definitions [7]. Participants were asked, "On average, how many days per week do you engage in moderate to strenuous exercise?" (Per AHA guidelines, moderate exercise intensity is 50% to about 70% of your maximum heart rate, and strenuous exercise intensity is 70% to about 85% of your maximum heart rate). Intensity and frequency were self-reported, and we used days per week of moderate-to-strenuous activity as the primary exposure. Duration (minutes per session) was not collected. For analysis, respondents were stratified based on symptom status: those reporting any history of SERMS and those without. The survey supporting this study is available from the corresponding author upon reasonable request.

Ethical considerations

No financial compensation was given for participation. The Institutional Review Board granted an exemption under category 2(ii). Informed consent was obtained from all participants via an electronic consent invitation prior to survey commencement.

Statistical analyses

Categorical variables were described through absolute and relative frequencies. Continuous variables were presented as medians with interquartile ranges [first quartile (Q1)-third quartile (Q3)]. Hypotheses were tested without adjustments using Fisher’s exact test for categorical variables and the Mann-Whitney U test for continuous variables with non-normal distributions. All significance levels were set at p < 0 .05.

## Results

**Figure 1:**
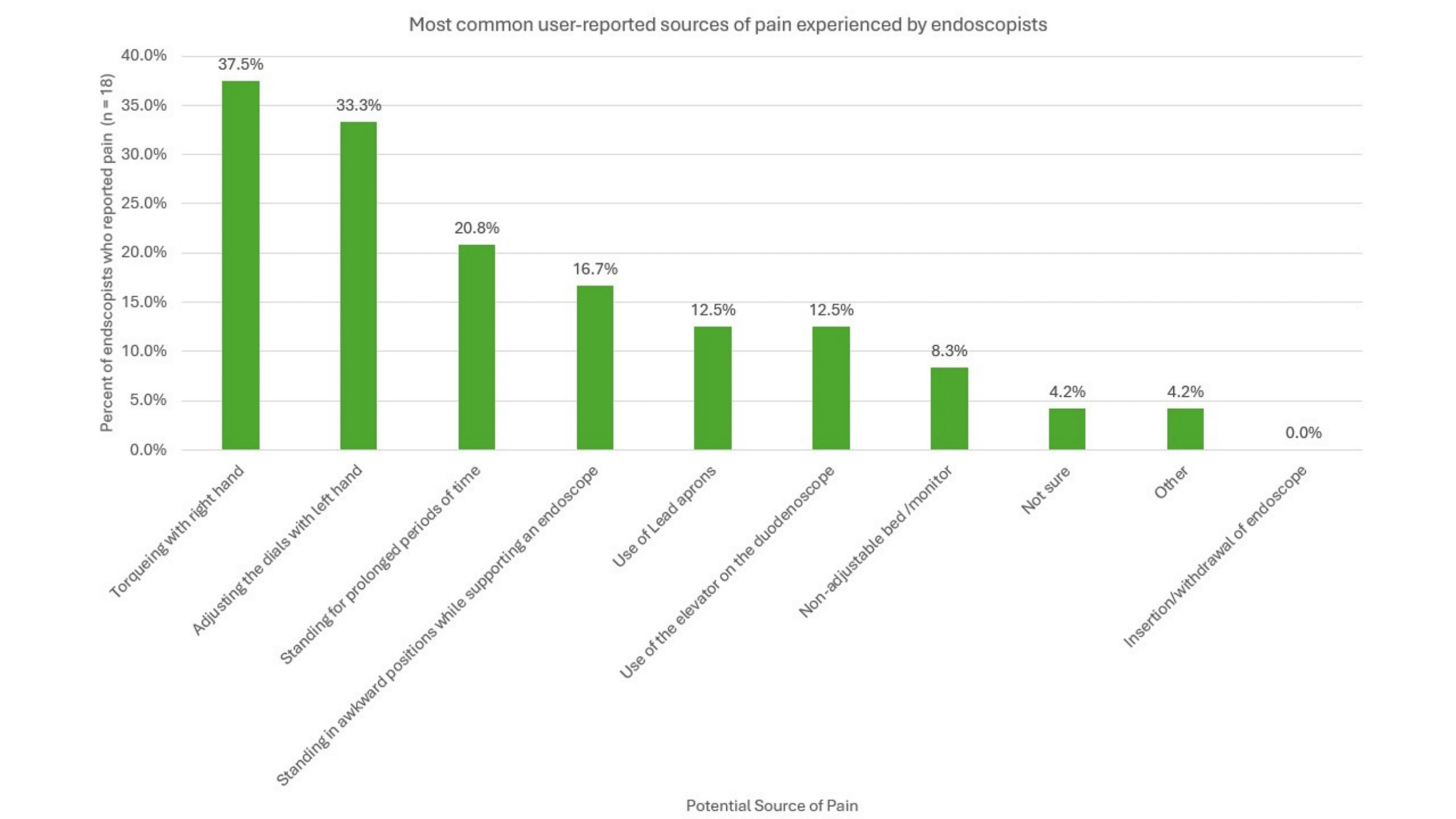
Most common user-reported source of pain experienced by endoscopists Bar graph depicting self-reported sources of self-reported endoscopy-related musculoskeletal symptoms among endoscopists. The most frequently reported source of pain was torqueing with the right hand, followed by adjusting dials with the left hand and standing for prolonged periods of time.

**Figure 2:**
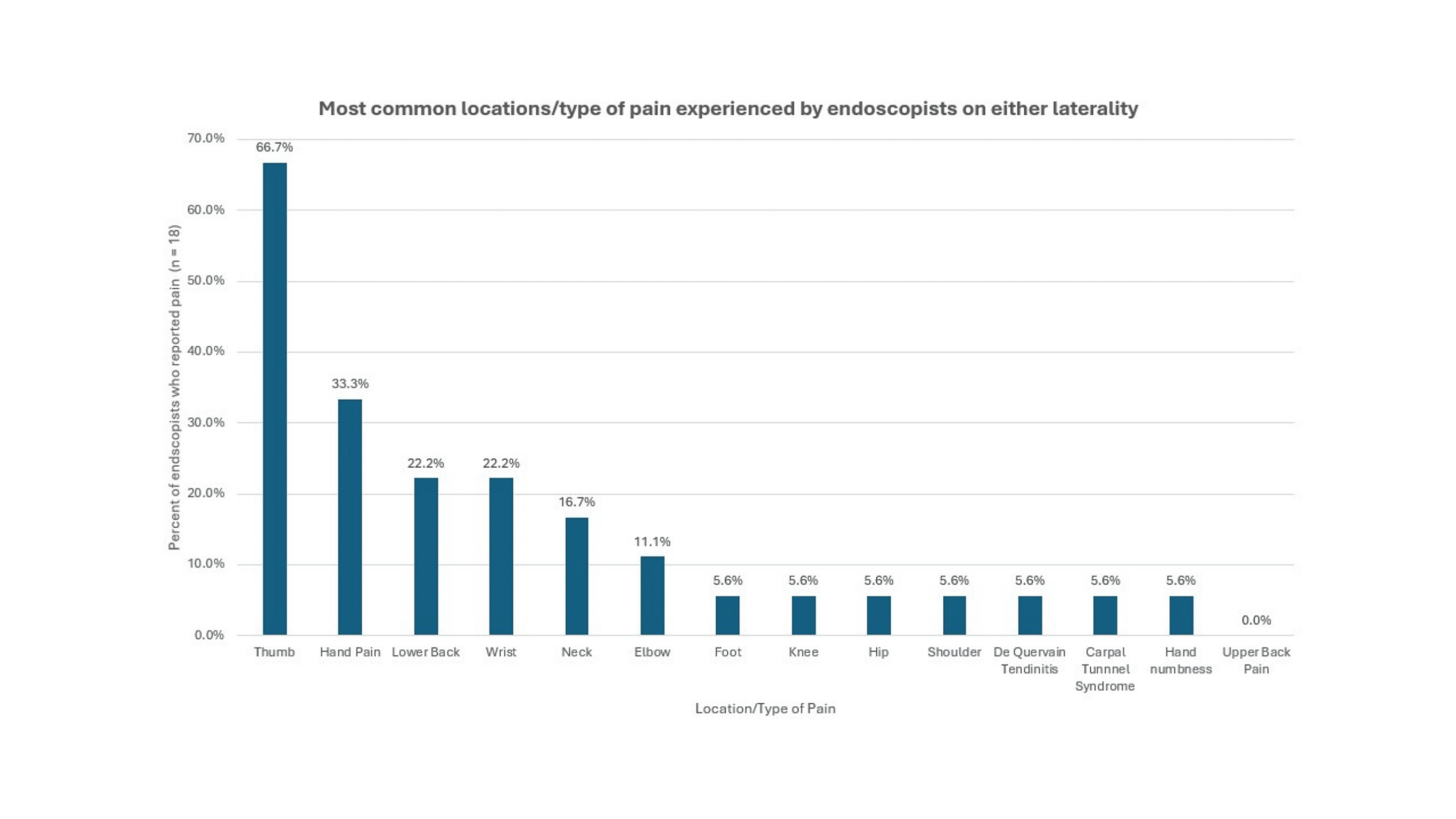
Most common locations/type of pain experienced by endoscopists on either laterality Bar graph showing the anatomic locations of pain experienced by endoscopists. The most common site of pain was the thumb, followed by the hand, lower back, and wrist.

A total of 50 participants were invited to complete the survey, including gastroenterologists, hepatologists, and gastroenterology fellows. Twenty-five (50%) of the participants completed the survey. Data regarding why some did not participate were not collected. At baseline, there were a total of 18 (72%) males and seven females (28%) who completed the survey. Overall, the median age of participants was 37.5 (34.25-40) years old, excluding three subjects who preferred not to answer. The median number of years of endoscopy experience (including fellowship years) was six (4-9). The median number of respondents engaged in moderate-to-strenuous exercises per week was three (3-4). Overall, 18 (72%) participants reported SERMS, while 7 (28%) reported no history of SERMS (Table 1). The median body mass index (BMI) was significantly greater in the SERMS group compared to the no-SERMS group (25.8 vs. 22.4, U = 26, p = 0.014). The most reported tasks associated with pain were torquing, adjusting dials, and standing for prolonged periods (Figure 1). The thumb was the most frequent location of pain among respondents, followed by the hand, lower back, and wrist (Figure 2).

**Table 1:** Characteristics of endoscopists Continuous and unadjusted overall variables are presented here. Localized subgroup analyses utilizing dichotomized variables are described separately within the text. ^ Three subjects in the SERMS group preferred not to answer. * Two subjects in the SERMS group preferred not to answer. SERMS: Self-reported endoscopy-related musculoskeletal symptoms; FET: Fisher’s exact test; U: Mann-Whitney U statistic

	SERMS (n = 18)	No SERMS (n = 7)	Total (n = 25)	Test statistic/method	p-value
Gender					
Male [n (%)]	15 (83.3)	3 (42.9)	18 (72.0)	FET	0.066
Female [n (%)]	3 (16.7)	4 (57.1)	7 (28.0)		
Age, median (Q1-Q3), years^	36 (34-39.5)	38 (36-43.5)	37.5 (34.25-40)	U = 40	0.395
Body Mass Index in kg/m^2^, median (Q1-Q3)*	25.8 (24.4-28.3)	22.4 (21.3-22.9)	24.4 (22.5-27.2)	U = 26	0.014
Years of experience, including fellowship (Q1-Q3)	5 (4-9)	8 (5-12)	6 (4-9)	U = 54.5	0.631
Days per week engaged in strenuous exercise (Q1-Q3)	3.5 (2-4.75)	4 (3-6.5)	4 (3-5)	U = 39.5	0.155
Approximate number of procedures performed in a week reported by respondent [n (%)]					
10-20	3 (16.7)	3 (42.9)	6 (24.0)	FET	0.298
20-30	7 (38.9)	1 (14.3)	8 (32.0)	FET	0.362
30-40	4 (22.2)	2 (28.6)	6 (24.0)	FET	> 0.999
40-50	2 (11.1)	1 (14.3)	3 (12.0)	FET	> 0.999
50-60	2 (11.2)	0 (0.0)	2 (8.0)	FET	> 0.999

While overall rates of SERMS did not significantly differ by baseline characteristics (Table 1), specific subgroup analyses revealed localized associations based on age, exercise habits, and years of experience. Participants were divided into younger (≤ 38 years, n = 11) and older (> 38 years, n = 11) groups based on median age. Younger gastroenterologists reported a significantly higher prevalence of hand pain compared to older ones (5 [45%] vs. 0 [0%], respectively, p = 0.035). However, there was no significant association between age and thumb pain (8 [73%] younger vs. 3 [27%] older, p = 0.086) or lower back pain (1 [9%] younger vs. 3 [27%] older, p = 0.311). Additionally, there was no significant difference in median age (as a continuous variable) between participants reporting no SERMS (median age = 38 years, n = 7) and those reporting any SERMS (median age = 36 years, n = 15) (U = 40, p = 0.395).

We also explored the effect of experience on SERMS. Participants were categorized into two groups: early-career gastroenterologists (six or fewer years of practice, including fellowship years, n = 13) vs. established gastroenterologists (more than six years of practice, including fellowship years, n = 12) based on median years of practice in our sample, as no specific threshold for injury risk has been established in the literature. Early-career gastroenterologists reported a significantly higher prevalence of thumb pain (9 [69%] vs. 3 [25%], p = 0.047) and hand pain (6 [46%] vs. 0 [0%], p = 0.015).

To measure the effect of exercise on SERMS and anatomic location, participants were sorted into two groups: those who engaged in moderate-to-strenuous exercise fewer than half the week (0-3 days/week, n = 12) vs. more than half the week (4-7 days/week, n = 13) based on median days per week of exercise in our sample, as no specific threshold for injury risk has been established in the literature. Our analysis identified a significant baseline difference in BMI between the exercise cohorts (U = 26.0, p = 0.014). Gastroenterologists who exercised fewer than half the week (0-3 days/week) reported a significantly higher prevalence of thumb pain compared to those who exercised more than half the week (9 [75%] vs. 3 [23%], p = 0.017). Hand pain was reported by 5 (42%) respondents who exercised fewer than half the week, compared to 1 (8%) respondent among those who exercised more than half the week, but this association was not significant (p = 0.155). There was no significant difference in the types of exercise performed by individuals with and without a history of SERMS using Fisher's exact test (Table 2). Additionally, there was no significant difference in the types of exercise performed by individuals who engaged in moderate-to-strenuous exercise at least four days per week compared to those who exercised fewer than four days per week (Appendix Table 3). Exercise frequency, when treated as a continuous variable, was higher in the no SERMS group, but this difference did not reach statistical significance (SERMS group: median 3.5 [2-4.75] days/week; no SERMS group: median 4 [3-6.5] days/week; U = 39.5, p = 0.155).

**Table 2:** Exercise modalities and their associations with SERMS SERMS: self-reported endoscopy-related musculoskeletal symptoms

Exercise Modality	SERMS (n = 18), n (%)	No SERMS (n = 7), n (%)	p-value
Resistance/Weight Training	12 (66.7%)	5 (71.4%)	> 0.999
Bicycling	8 (44.4%)	4 (57.1%)	0.673
Running	7 (38.9%)	5 (71.4%)	0.202
Other	5 (27.8%)	0 (0.0%)	0.274
Structured Classes	4 (22.2%)	2 (28.6%)	> 0.999
Swimming	1 (5.6%)	1 (14.3%)	0.490
Yoga / Stretching	1 (5.6%)	3 (42.9%)	0.053

We also explored the effect of procedure frequency on SERMS location. Subjects were stratified into either the moderate-volume (10-30 procedures/week, n = 14) or high-volume (31-60 procedures/week, n = 11) group based on median weekly procedural load, as no specific threshold for injury risk has been established in the literature. There was no significant association between procedure load (moderate vs. high volume) and the prevalence of thumb pain (6 [43%] vs. 6 [55%], p = 0.695), hand pain (2 [14%] vs. 4 [36%], p = 0.350), or low back pain (4 [29%] vs. 0 [0%], p = 0.105).

Finally, participants were also categorized based on prior ergonomics training: those with ergonomics training (n = 10) and those without ergonomics training (n = 15). There was no significant association between a history of ergonomics training and the prevalence of thumb or hand pain. Thumb pain was reported in 7 (70%) respondents with ergonomics training compared to 5 (33%) without training (p = 0.111), and hand pain was reported in 4 (40%) respondents with training versus 2 (13%) without training (p = 0.175).

## Discussion

Overall, endoscopists face high rates of musculoskeletal injuries related to performing endoscopy [1]. Specifically, we focused on the relationship between exercise habits and SERMS, as this has yet to be investigated systematically.

Our cohort had a median age in the late thirties (37.5 years) and a median of six years of endoscopy experience, including fellowship. Nearly three-quarters (72%) of participants reported SERMS, which is slightly higher than the overall rate of ERIs of 57.7% (95% confidence interval, 48.8-66.1) among endoscopists cited in existing literature [1]. Participants reported engaging in moderate-to-strenuous exercise about half of the days per week. Our study suggests that moderate-to-strenuous exercise for more than half the days of the week, compared to less than half the week, may be associated with a lower prevalence of self-reported thumb pain from performing endoscopy. This may be because endoscopy requires sustained pinch grip and fine motor control at the control section of the endoscope. Perhaps regular exercise may improve muscle conditioning and neuromuscular endurance that could affect the thumb.

Hand pain was significantly associated with younger age and fewer years of endoscopy experience. This may reflect an adjustment period for less experienced endoscopists as they develop skill and familiarity with proper technique. Perhaps targeted ergonomic guidance during the first several years of practice may be beneficial to health. The age-related association appears limited to hand pain rather than broadly any musculoskeletal injury (thumb, hand, back, etc.).

In contrast to prior studies with larger survey data, we did not observe a positive volume-injury relationship in our cohort [2]. We compared endoscopists who performed 10-30 vs. 31-60 procedures per week. The lack of statistical significance may be due to the sample size, age, experience level of the participants, or individual factors (ergonomic technique or physical conditioning). This may limit the ability to detect modest associations. We also did not observe any association between prior ergonomics training and thumb or hand pain. However, this null result should be interpreted with caution, as any ergonomics training done was not done at the same institution as the survey, and we did not evaluate the details of any participant's prior training.

Because research on ergonomics in endoscopy is a newly evolving field, data are limited. So far, guidelines suggest that procedure volume, years in practice, female gender, and small hand size are risk factors for ERIs, but research on safe practices and preventive measures remains limited [1,8]. The American Society of Gastrointestinal Endoscopy recommends ergonomics education and practical interventions, such as microbreaks, neutral bed positioning, and antifatigue mats, but does not comment on physical exercise as a prevention strategy [1]. The American College of Occupational and Environmental Medicine guidelines recommend exercise prescriptions for the prevention of work disability due to musculoskeletal injuries in general. Our findings suggest that perhaps those general principles could apply to SERMS and suggest that further research into the optimal type, intensity, and frequency of exercise could shed more light on endoscopist occupational health [4].

BMI and obesity have been associated with increased occupational musculoskeletal injury in prior research [9]. We observed a significant difference in BMI between participants with and without SERMS. Thus, BMI may be a plausible confounder affecting the observed association between exercise frequency and thumb pain. This may also reflect healthy user bias, which is when the tendency for individuals who adopt health‑promoting behaviors (exercise) differs systematically (e.g., BMI) from non‑adopters [10]. Given our sample size and limited event counts, performing multivariable adjustment to isolate the independent effects of BMI and exercise was not feasible without risking overfitting and model instability; this methodological constraint is well recognized in prior research on musculoskeletal injuries associated with endoscopy [11]. Thus, we transparently present unadjusted associations while explicitly acknowledging this limitation and avoiding drawing overly general conclusions. Moreover, there is a dearth of studies examining the relationship between BMI and SERMS specifically, and physician‑focused data on BMI and occupational musculoskeletal injury are limited. Future studies should account for BMI or perhaps investigate any association between BMI and SERMS. This study was also subject to additional limitations, including a modest response rate, single-center design/limited generalizability, and potential temporality and recall bias. While the cross-sectional nature of this study limits our ability to establish a temporal relationship between the timing of the SERMS and exercise habits, these data provide a valuable baseline for the association between physical activity and occupational injury. To account for the limited sample size, we utilized conservative statistical methods, like Fisher’s Exact Test, as it is specifically designed for smaller datasets, and it provides a calculation of the probability rather than approximations that are more appropriate for larger datasets. These findings should be considered hypothesis-generating and preliminary rather than causative or practice-changing at this time. While prior ERI research focused on procedural load or equipment, our study shifts the focus towards an endoscopist's physical conditioning and occupational health. Despite our small cohort, a statistically significant association between reduced prevalence of SERMS and more frequent moderate-to-strenuous exercise emerged using conservative methods that may warrant further investigation in larger, prospective trials.

## Conclusions

Our study’s findings signal potential associations between exercise, age, and experience level that warrant deeper investigation with larger and more targeted prospective studies. The early observation that more frequent moderate-to-strenuous exercise may be associated with a reduced prevalence of thumb SERMS is a novel one that is hypothesis-generating and warrants confirmation in larger samples and prospective trials. Using the known muscle groups involved in performing endoscopy, a multidisciplinary, structured, guided exercise program may be beneficial for gastroenterologists across experience levels. Further studies that delve into the types of exercise and muscle groups involved can pave the way for improved endoscopist occupational health.

## Appendices

**Table 3:** Comparison of specific exercise modalities between individuals who engage in frequent and infrequent moderate-to-strenuous exercise There was no significant difference in the types of exercise performed by individuals or frequency of exercise. Data are presented as percentages of the respective frequency group. P-values determined by Fisher's exact test.

Exercise Modality	Frequent (≥ 4 days/week) (n = 13), n (%)	Infrequent (≤ 3 days/week) (n = 12), n (%)	p-value
Weights	8 (61.5%)	5 (41.7%)	0.434
Resistance Training	7 (53.8%)	3 (25.0%)	0.226
Running	7 (53.8%)	5 (41.7%)	0.695
Bicycling	7 (53.8%)	4 (33.3%)	0.428
Structured Classes	3 (23.1%)	3 (25.0%)	> 0.999
Yoga/Stretching	2 (15.4%)	2 (16.7%)	> 0.999
Other	4 (30.8%)	1 (8.3%)	0.322
